# RGS9 Knockout Causes a Short Delay in Light Responses of ON-Bipolar Cells

**DOI:** 10.1371/journal.pone.0027573

**Published:** 2011-11-11

**Authors:** Rolf Herrmann, Bowa Lee, Vadim Y. Arshavsky

**Affiliations:** Albert Eye Research Institute, Duke University, Durham, North Carolina, United States of America; Oregon Health & Science University, United States of America

## Abstract

RGS9 and R9AP are components of the photoreceptor-specific GTPase activating complex responsible for rapid inactivation of the G protein, transducin, in the course of photoresponse recovery from excitation. The amount of this complex in photoreceptors is strictly dependent on the expression level of R9AP; consequently, the knockouts of either RGS9 or R9AP cause comparable delays in photoresponse recovery. While RGS9 is believed to be present only in rods and cones, R9AP is also expressed in dendritic tips of ON-bipolar cells, which receive synaptic inputs from photoreceptors. Recent studies demonstrated that knockouts of R9AP and its binding partner in ON-bipolar cells, RGS11, cause a small delay in ON-bipolar cell light responses manifested as a delayed onset of electroretinography b-waves. This led the authors to suggest that R9AP and RGS11 participate in regulating the kinetics of light responses in these cells. Here we report the surprising finding that a nearly identical b-wave delay is observed in RGS9 knockout mice. Given the exclusive localization of RGS9 in photoreceptors, this result argues for a presynaptic origin of the b-wave delay in this case and perhaps in the case of the R9AP knockout as well, since R9AP is expressed in both photoreceptors and ON-bipolar cells. We also conducted a detailed analysis of the b-wave rising phase kinetics in both knockout animal types and found that, despite a delayed b-wave onset, the slope of the light response is unaffected or increased, dependent on the light stimulus intensity. This result is inconsistent with a slowdown of response propagation in ON-bipolar cells caused by the R9AP knockout, further arguing against the postsynaptic nature of the delayed b-wave phenotype in RGS9 and R9AP knockout mice.

## Introduction

At the first step of visual processing in the vertebrate retina, photoreceptors convey light-evoked signals to bipolar cells [1]. Both cell types rely on G protein-mediated signaling pathways for generating their light-responses. Accordingly, the amplitude and kinetics of photoresponses in rods and cones are tightly controlled by the rates at which the G protein, transducin, is activated by the GPCR receptor, rhodopsin, and inactivated through the mechanism of GTP hydrolysis (see [2], [3] for comprehensive reviews and [4], [5] for more recent updates). While transducin has the intrinsic ability to hydrolyze bound GTP, the rate of this reaction is slow and, in photoreceptors, it is accelerated roughly 100-fold by the GTPase activating protein, RGS9 [6], [7], [8].

In rods and cones, RGS9 exists as a complex with its obligatory Gβ5 subunit [9] and the anchor protein, R9AP [10]. R9AP is a multi-functional protein. In addition to tethering RGS9·Gβ5 on the surface of photoreceptor discs, R9AP enhances the ability of RGS9·Gβ5 to activate transducin GTPase [11], [12], directs RGS9·Gβ5 to outer segments [13], [14], and protects RGS9·Gβ5 from intracellular proteolysis, ultimately setting the expression level of the entire RGS9·Gβ5·R9AP complex. The protective role of R9AP was established by demonstrating that R9AP knockout causes complete elimination of RGS9 from photoreceptors [15], whereas R9AP overexpression in rods increases the amounts of RGS9 and Gβ5 as well [16].

While RGS9 in the retina was found to be expressed exclusively in photoreceptors (e.g. [6], [17], [18]), a distinct fraction of R9AP is present in dendritic tips of ON-bipolar cells [19], [20], where it stabilizes another RGS protein complex, RGS11·Gβ5 [19], [21], [22]. This complex is thought to contribute to rapid inactivation of G_o_, the G protein implicated in mediating light signaling in these cells [23], [24]. Light signals in ON-bipolar cells are triggered by a reduction in the glutamate release from photoreceptors, which is closely monitored by the mGluR6 receptors located in dendritic tips of ON-bipolar cells [25], [26]. Downstream from mGluR6 are the TRPM1 channels [27], [28], [29], which open in response to the light-induced suppression of mGluR6 activity. The current working model (e.g. [24], [30], [31]) suggests that the signal between mGluR6 and TRPM1 is carried by G_o_, which undergoes a rapid activation/inactivation cycle, catalyzed by mGluR6 and RGS proteins, respectively. In this model, the light-dependent cessation of mGluR6 stimulation by glutamate leads to rapid TRPM1 opening, with response kinetics at least not slower than the G_o_ inactivation rate.

In general agreement with this model, the knockouts of both RGS11 and R9AP have been shown to cause a delay in ON-bipolar cell light responses, documented by recording electroretinography (ERG) b-waves [20], [32], [33]. However, this b-wave delay is very small, typically under ∼15 ms, which is an ∼1000-fold shorter time than is required for G_o_ to hydrolyze bound GTP in the absence of RGS proteins [34]. One explanation for such small effects of RGS11 and R9AP knockouts is that ON-bipolar cell dendrites contain another RGS protein complex, RGS7·Gβ5, anchored by the R9AP homolog, R7BP [17]. RGS7·Gβ5 may be sufficient for rapid G_o_ inactivation when RGS11·Gβ5 is absent [32], [33] and, in fact, RGS11 knockout is accompanied by an increase in the RGS7 content of the retina [32].

Here we report a surprising observation that the knockout of RGS9 causes a delay in ERG b-wave responses essentially identical to that observed in the R9AP knockout. Because RGS9 is expressed in photoreceptors only, this effect would be expected to have a presynaptic origin. Furthermore, because RGS9 expression in photoreceptors is strictly dependent on the expression of R9AP, this result also suggests that the identical b-wave delay in the R9AP knockout may be explained by the lack of RGS9 in rods and cones.

## Results

### R9AP expression in ON-bipolar cell dendrites and photoreceptor synaptic morphology are not affected by the RGS9 knockout

The goal of our study was to conduct a comprehensive comparison of ERG responses in *R9AP* ^−/−^ and *RGS9* ^−/−^ mice. This required two control experiments addressing: (1) whether the expression of R9AP in dendritic tips of ON-bipolar cells is affected by the RGS9 knockout, and (2) whether either R9AP or RGS9 knockout affects the morphology of the photoreceptor-to-ON-bipolar cell synapses.

**Figure 1:**
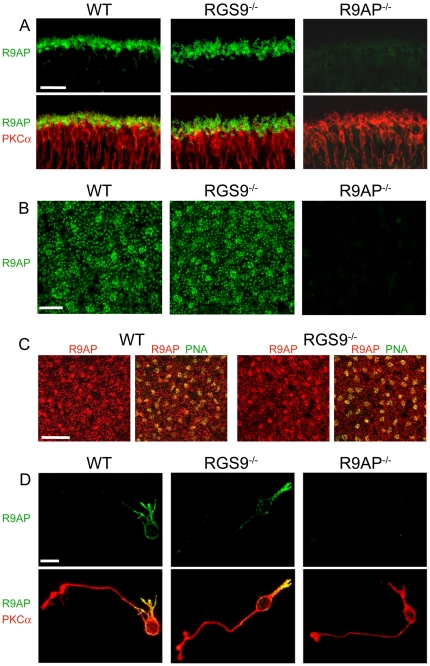
R9AP expression in ON-bipolar cells of WT and *RGS9* ^−/−^ retinas. (**A**) Co-immunostaining of retina cross-sections from WT, *RGS9* ^−/−^ and *R9AP* ^−/−^ mice for R9AP (green) and the rod ON-bipolar cell marker, PKCα (red). Shown are the outer plexiform layers of each section. Scale bar: 25 µm. (**B**) Single confocal sections through the outer plexiform layers of whole mount retinas of WT, *RGS9* ^−/−^ and *R9AP* ^−/−^ mice immunostained for R9AP. We observed no notable differences in the pattern of R9AP immunostaining between *RGS9* ^−/−^ (40 frames from 6 retinas) and WT controls (30 frames from 6 retinas). Scale bar: 10 µm. (**C**) Single confocal sections through the outer plexiform layers from whole mount retinas of WT and *RGS9* ^−/−^ mice co-immunostained for R9AP (red) and peanut agglutinin (PNA, green) labeling cone pedicles in the outer plexiform layer. Scale bar: 25 µm. (**D**) Rod ON-bipolar cells dissociated from WT, *RGS9* ^−/−^ and *R9AP* ^−/−^ retinas co-immunostained for R9AP and PKCα. Scale bar: 10 µm.

The localization of R9AP in ON-bipolar cells of *RGS9* ^−/−^ and WT mice was first examined in retina cross-sections. Figure 1a shows that R9AP immunostaining in the outer plexiform layer (where synapses between photoreceptors and bipolar cells are located), was unaffected by the RGS9 knockout. Most staining was observed at the tips of bipolar cell dendrites of rod ON-bipolar cells, stained with a specific marker of these cells, PKCα. The specificity of R9AP immunostaining in this and all subsequent experiments was verified using the retinas from R9AP knockout mice. The similarity in the R9AP staining patterns of outer plexiform layers in *RGS9* ^−/−^ and WT mice was particularly well-appreciated in immunostained retina whole mounts (Figure 1b), where the dendritic tips of rod ON-bipolar cells appear as scattered puncta. This view was also most instructive for visualizing cone-to-ON-bipolar cell synapses, which appear as patches colocalized with the cone-specific marker, peanut agglutinin (Figure 1c).

We also analyzed R9AP immunostaining in dissociated rod ON-bipolar cells from *RGS9* ^−/−^ and WT mice (Figure 1d). In both cases, R9AP was found throughout the dendrites and to a lesser degree in the cell soma. Such a loss of protein localization to dendritic tips in dissociated rod bipolar cells was previously documented for another synaptic protein, mGluR6 [35], and may be interpreted as a consequence of lacking presynaptic cell interactions. However, we observed no systematic difference in the R9AP staining patterns of fifteen dissociated *RGS9* ^−/−^ and twelve dissociated WT cells retaining overall normal morphology.

**Figure 2:**
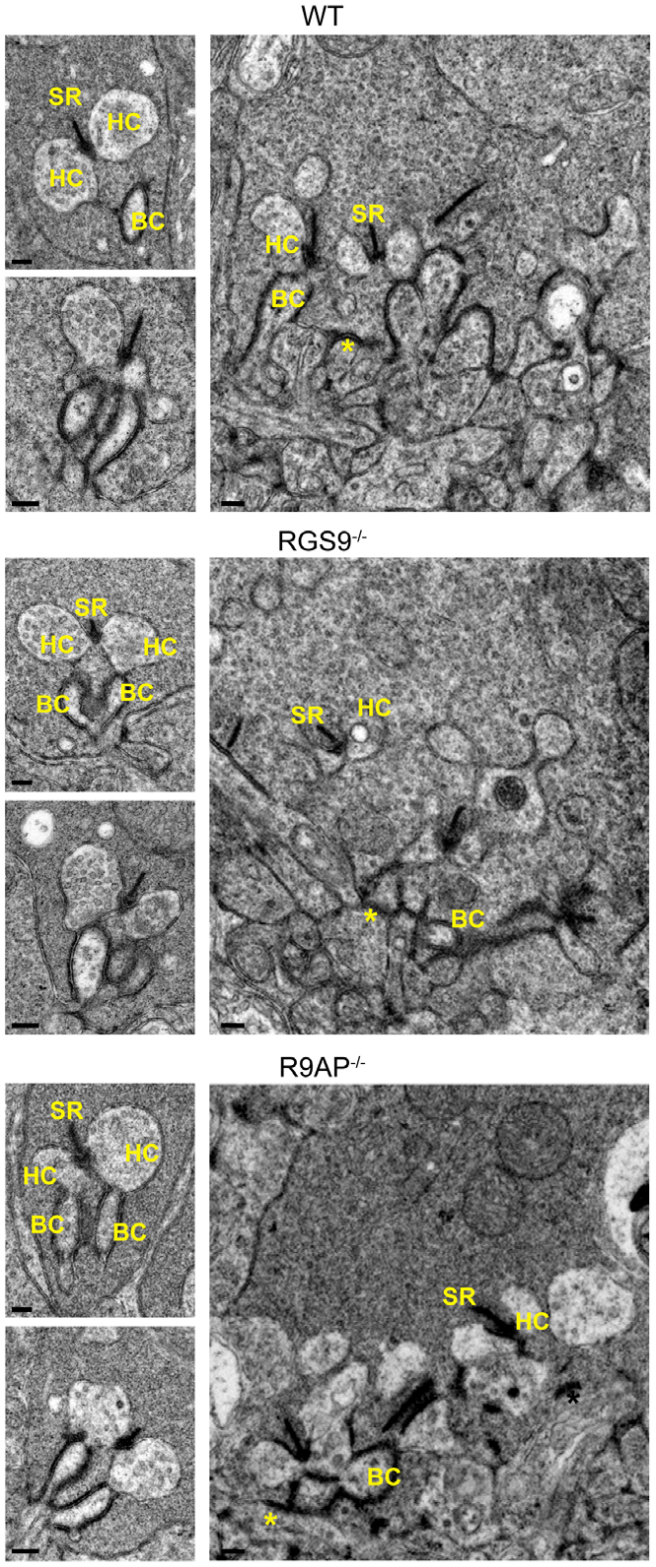
Electron microscopy analysis of rod and cone synaptic terminals in WT, *RGS9* ^−/−^ and *R9AP* ^−/−^ mice. Shown are representative electron micrographs of cross-sections through rod spherules (smaller images on the left) and through the base of cone pedicles (right). At least 60 rod spherules and 10 cone pedicles were analyzed for each mouse type. Abbreviations are: SR – synaptic ribbon, HC – horizontal cell process, BC – bipolar cell dendrite. Asterisks indicate flat synaptic contacts at the cone terminals. Scale bars: 200 nm.

We next examined the ultrastructure of rod and cone synaptic terminals in *R9AP* ^−/−^ and *RGS9* ^−/−^ mice using electron microscopy (Figure 2). In both mouse types, rod synapses appeared normal, as judged from the structure of synaptic ribbons and invaginating postsynaptic processes of horizontal and bipolar cells. Similarly, pre- and postsynaptic structural elements of cone synaptic terminals of these mice appeared normal. While we are not aware of any similar published data on the RGS9 knockout, the observation of normal synaptic morphology in *R9AP* ^−/−^ mice is entirely consistent with the recent report by Jeffrey et al. [20]. Taken together, the data shown in Figs. 1 and 2 did not reveal any notable changes in R9AP localization in the outer plexiform layer of *RGS9* ^−/−^ mice or any morphological abnormalities in either knockout mouse type.

### Characterization of ERG responses in R9AP and RGS9 knockout mice

Light responses of *R9AP* ^−/−^ and *RGS9* ^−/−^ mice were analyzed by electroretinography. ERGs are field potentials recorded at the cornea, which reflect the cumulative light-evoked activity of several types of retinal neurons. A typical ERG response has two phases: a- and b-waves. The a-wave is a negative deflection immediately following the light stimulus, which primarily originates from suppression of the circulating dark current in rod and cone outer segments. The subsequent positive deflection, called the b-wave, originates primarily from the light-induced depolarizing currents in ON-bipolar cells [36], [37].

**Figure 3:**
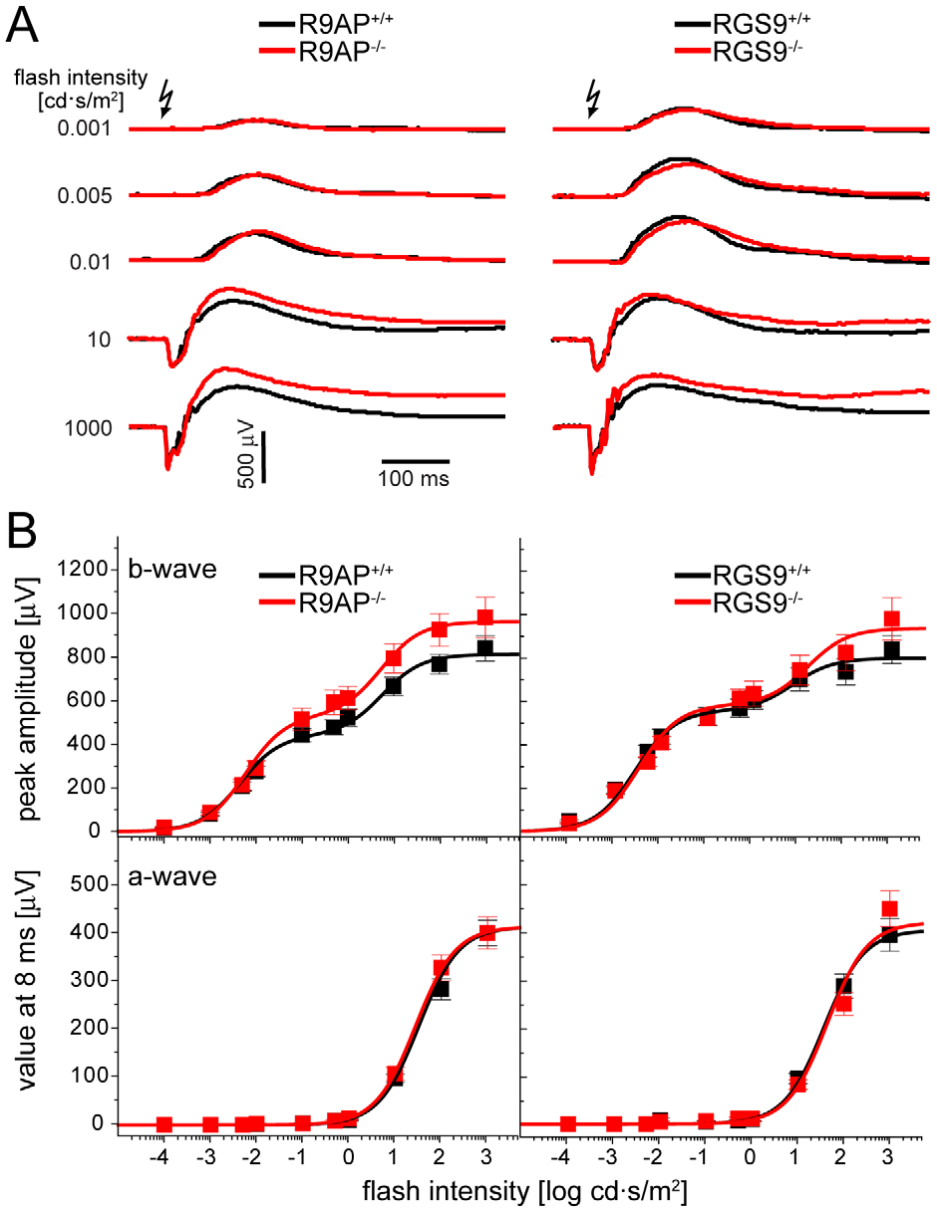
ERG responses of *R9AP* ^−/−^ and *RGS9* ^−/−^ mice. (**A**) ERG recordings from *R9AP* ^−/−^ or *RGS9* ^−/−^ mice (red) and their corresponding WT littermates (black) were averaged from all recordings evoked by a given flash intensity. Arrows indicate the time when light flash was applied. (**B**) Stimulus-response curves of b-wave amplitudes (upper panel) and a-wave values measured at 8 ms after the flash, a time point preceding the b-wave onset (lower panel). Data for each knockout mouse are shown in red, data for WT littermates are shown in black. Data points (mean ± SEM) were fitted using Equation 1 (Materials and Methods); fitting parameters are summarized in Table 1. The data were averaged from 13 eyes of *R9AP* ^−/−^; 13 eyes of *R9AP* ^+/+^; 7 eyes of *RGS9* ^−/−^; and 7 eyes of *RGS9* ^+/+^ mice.

Figure 3a shows averaged ERG responses of *R9AP* ^−/−^, *RGS9* ^−/−^ and WT littermate mice evoked by light flashes of different intensities. Complete stimulus-response curves for both a- and b-wave responses are shown in Figure 3b, while the summary of fitting parameters is presented in Table 1. Overall, the ERG responses of *R9AP* ^−/−^ and *RGS9* ^−/−^ mice were very similar to those recorded from the corresponding WT controls. The a-waves were virtually identical under all tested conditions. The b-waves displayed a trend of increased amplitudes, particularly at brighter flash intensities, an effect noted in previous studies [8], [20]. However, a paired independent t-test did not reveal this trend to be statistically significant. For example, at the flash intensity of 1000 cd·s/m^2^, p-values for the difference between b-wave amplitudes in knockout and WT littermates were 0.23 and 0.19 for *RGS9* ^−/−^ and *R9AP* ^−/−^ mice, respectively.

**Table 1:** A summary of fitting parameters obtained from the analysis of ERG b-wave and a-wave stimulus-response curves in Figure 3B.

	*I_0.5,1_* [cd·s/m^2^]	*R_max,1_*	*I_0.5,2_* [cd·s/m^2^]	*R_max,2_*
**a-wave**				
***RGS9*** **^+/+^**	36.9±0.8	406±2	---	---
***RGS9*** **^−/−^**	43.5±5.8	422±20	---	---
***R9AP*** **^+/+^**	38.7±1.1	409±2	---	---
***R9AP*** **^−/−^**	27.6±0.3	413±1	---	---
**b-wave**				
***RGS9*** **^+/+^**	0.0027±0.0002	555±8	7.2±1.6	241±11
***RGS9*** **^−/−^**	0.0032±0.0003	581±11	14.7±1.1	351±17
***R9AP*** **^+/+^**	0.0044±0.0003	445±7	5.6±0.7	373±9
***R9AP*** **^−/−^**	0.0059±0.0004	535±9	5.3±0.6	433±10

The data were averaged from 13 eyes of *R9AP* ^−/−^; 13 eyes of *R9AP* ^+/+^; 7 eyes of *RGS9* ^−/−^; and 7 eyes of *RGS9* ^+/+^ mice and were fitted using *Equation 1* (see Materials and Methods); *I_0.5,1_* and *I_0.5,2_* are half-saturating flash intensities of rod- and cone-driven responses, *R_max,1_* and *R_max,2_* are the corresponding maximal response amplitudes. Fitting parameters are given as mean ± SEM.

### R9AP and RGS9 knockout mice display a distinct delay in the rising phase of their b-wave responses but not a reduction in response slope

**Figure 4:**
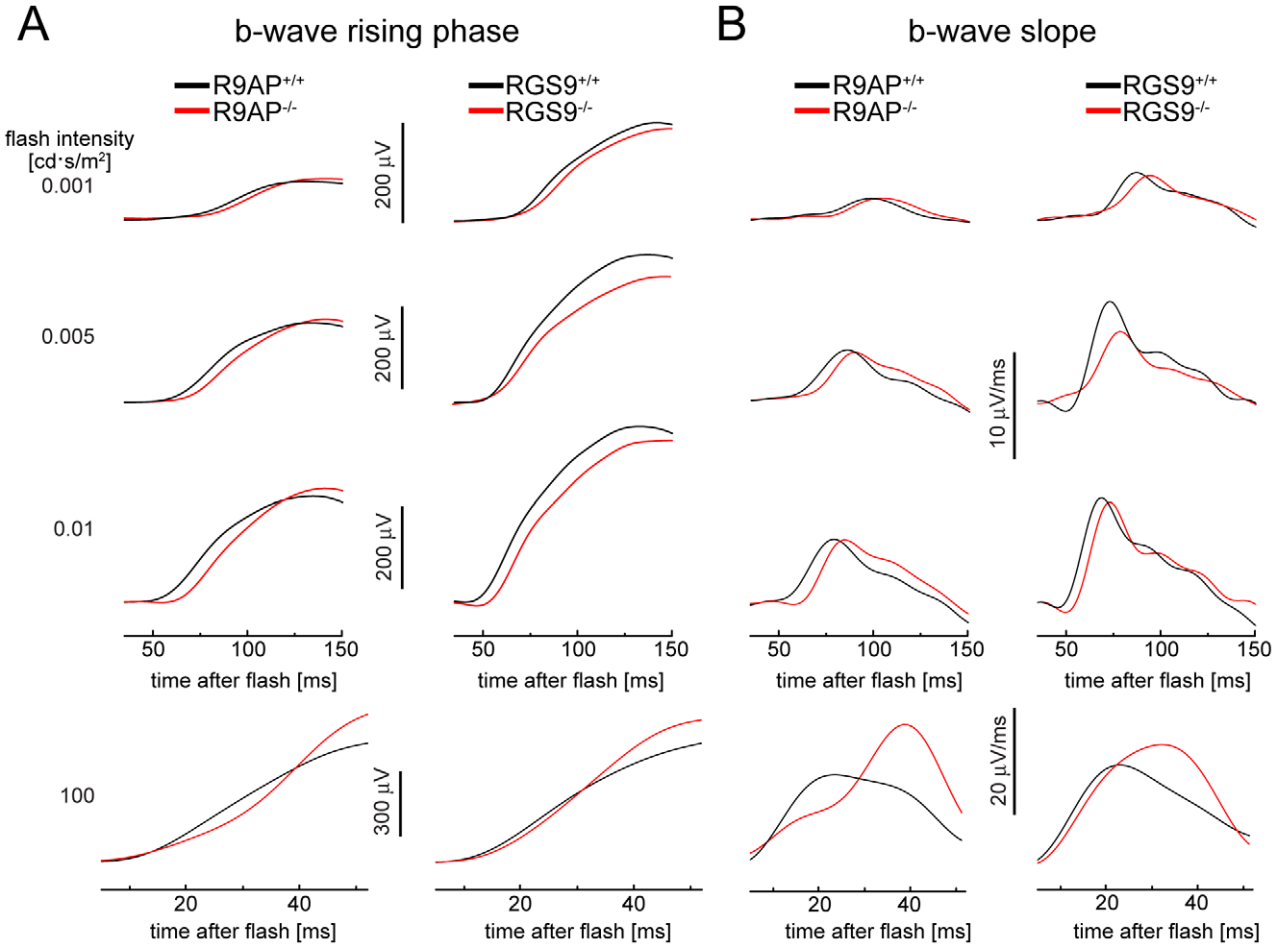
The effects of R9AP and RGS9 knockouts on the b-wave rising phase. (**A**) Examples of averaged b-wave responses from *R9AP* ^−/−^ and *RGS9* ^−/−^ (red) and WT (black) mice shown on a shorter time scale than in Figure 3A to illustrate differences in the rising phase kinetics. Traces were filtered to remove oscillatory potentials and the a-wave was additionally subtracted from the 100 cd·s/m^2^ flash responses. (**B**) The first derivatives of the traces in (A) used to calculate maximal slopes of the b-wave rising phase and times required to reach the maximal slope.

To conduct a detailed analysis of the b-wave rising phase kinetics, we re-plotted the data from Figure 3a on a shorter timescale (Figure 4a). In this analysis, we also removed the oscillatory potentials (high frequency wavelets originating from inner retina activity that superimpose the rising b-wave) and subtracted a-waves where applicable (see Materials and Methods for details). On this timescale, it is easy to see the previously reported delay in b-wave rising phase caused by the R9AP knockout [20]. However, it is also evident that a comparable delay is present in *RGS9* ^−/−^ mice. This delay can be further illustrated by plotting first derivatives of b-wave responses [20], which reflect the slopes of b-wave rising phases (Figure 4b). Note that the small differences in response amplitudes and maximal slopes between the two WT controls are most likely explained by differences in genetic backgrounds.

**Figure 5:**
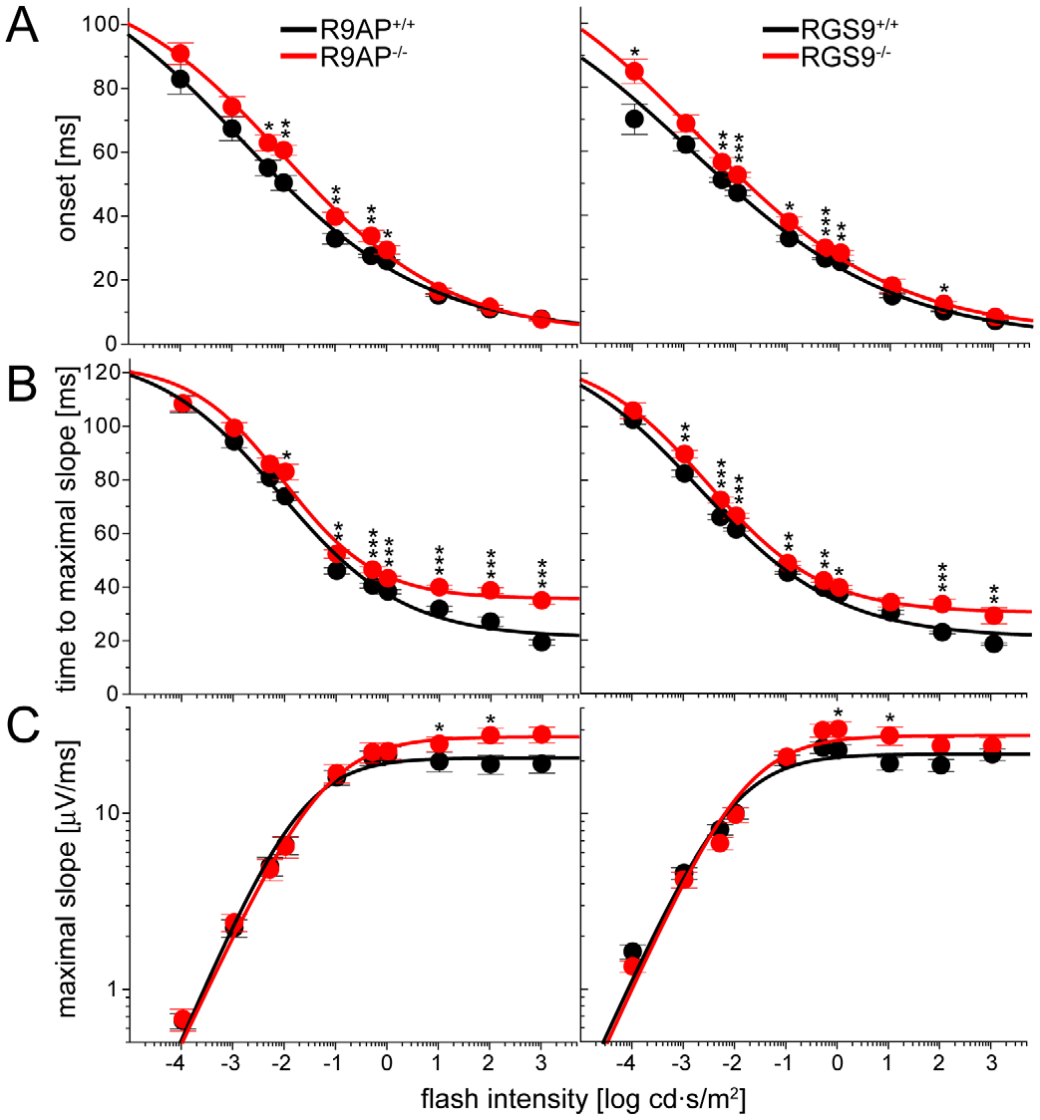
Plots of three parameters characterizing the rising phases of b-waves as functions of flash intensity. (**A**) Time of b-wave onset. (**B**) Time required to reach the maximal slope of the rising b-wave. (**C**) The value of maximal b-wave slope. The data in (A) and (B) were fitted by Equation 2 (fitting parameters are summarized in Tables 2 and 3, respectively); the data in (C) were fitted to Equation 3 (fitting parameters are summarized in Table 4). The statistical significance of the difference between the pairs of mean values obtained from knockout mice and their WT littermates was determined by a paired independent t-test, yielding p-values marked as follows: (*) p<0.05; (**) p<0.01; (***) p<0.001.

To analyze the kinetics of the b-wave rising phase in each knockout quantitatively, we calculated the mean values of three response parameters at multiple flash intensities (Figure 5): 1) the time of b-wave onset, 2) the time required to reach the maximal b-wave slope, and 3) the maximal value of the b-wave slope.

**Table 2:** A summary of fitting parameters for the analysis of ERG b-wave onset time as a function of flash intensity.

	*y_max_* [ms]	*n*	*k* [cd·s/m^2^]	*y_0_* [ms]
***RGS9*** **^+/+^**	119±13	0.22±0.02	0.0013±0.0011	1.4±1.1
***RGS9*** **^−/−^**	127±3	0.23±0.01	0.0017±0.0004	3.4±0.6
***R9AP*** **^+/+^**	123±5	0.25±0.01	0.0016±0.0005	3.4±0.8
***R9AP*** **^−/−^**	115±4	0.27±0.01	0.0095±0.0021	2.8±1.0

The data were fitted using *Equation 2* (see Materials and Methods); *y_max_* is the asymptotic maximal onset value, *n* is the Hill coefficient, and *k* and *y_0_* are fitting parameters (mean ± SEM).

**Table 3:** A summary of fitting parameters for the analysis of the time required to reach the maximal slope of b-wave rising phase as function of flash intensity.

	*y_max_* [ms]	*n*	*k* [cd·s/m^2^]	*y_0_* [ms]
***RGS9*** **^+/+^**	133±6	0.32±0.02	0.0019±0.0007	21.2±1.0
***RGS9*** **^−/−^**	127±2	0.39±0.01	0.0028±0.0003	30.4±0.3
***R9AP*** **^+/+^**	128±4	0.36±0.02	0.0081±0.0019	20.9±1.1
***R9AP*** **^−/−^**	124±8	0.48±0.03	0.0092±0.0014	35.5±0.9

The data were fitted using *Equation 2* (see Materials and Methods); *y_max_* is the maximal onset value, *n* is the Hill coefficient, and *k* and *y_0_* are fitting parameters (mean ± SEM).

**Table 4:** A summary of fitting parameters for the analysis of the maximal slope of ERG b-wave rising phase as function of flash intensity.

	*I_half_* [cd·s/m^2^]	*S_max_* [µV/ms]	*n*
***RGS9*** **^+/+^**	0.0085±0.0012	21.8±0.4	0.65±0.06
***RGS9*** **^−/−^**	0.0151±0.0026	27.7±0.5	0.65±0.06
***R9AP*** **^+/+^**	0.0209±0.0024	20.8±0.3	0.68±0.04
***R9AP*** **^−/−^**	0.0551±0.0029	27.4±0.2	0.63±0.02

The data were fitted using *Equation 3* (see Materials and Methods); *I_half_* is the half-saturating flash intensity, *S_max_* is the asymptotic value of the maximal slope at saturating flash intensities, and *n* is the Hill coefficient (mean ± SEM).

Both mice were characterized by an increase in the b-wave onset time observed at flash intensities up to 1 cd·s/m^2^ (Figure 5a). For instance, the average b-wave onset at the flash intensity of 0.005 cd·s/m^2^ was increased by 8 ms in *R9AP* ^−/−^ mice (p = 0.033) and by 6 ms in *RGS9* ^−/−^ mice (p = 0.003). This delay decreased with increasing flash intensity and became negligible at the three brightest flashes. This is why a-wave amplitudes evoked by bright flashes in *R9AP* ^−/−^ and *RGS9* ^−/−^mice were not increased compared to WT controls (Figure 3a), as may be expected from a delay in b-wave onset.

Both knockouts also delayed the time required for the b-wave to reach its maximal slope. However, this effect was most pronounced at bright flashes (Figure 5b). For instance, at the flash intensity of 1000 cd·s/m^2^, a 15 ms delay was observed in *R9AP* ^−/−^ mice (p = 9.7·10^−9^) and an 11 ms delay in *RGS9* ^−/−^ mice (p = 0.001). This delay is similar to that reported by Jeffrey et al. [20], who found that b-waves of *R9AP* ^−/−^ mice were delayed by an average of 13 ms at flash intensities ≥∼30 cd·s/m^2^.

Another parameter affected by both knockouts at brighter flash intensities over 1 cd·s/m^2^ was an increase in the maximal b-wave slope (Figure 5c). This parameter was not specifically analyzed by Jeffrey et al. [20]; however, a similar effect caused by the R9AP knockout could be seen in Figures 4 and 6 of their paper.

Taken together, our results demonstrate that the onset of the b-wave is similarly delayed in *R9AP* ^−/−^ and *RGS9* ^−/−^ mice. At least one response parameter, the time of b-wave onset or the time at which a b-wave reaches its maximal slope, was affected at all flash intensities tested in our study. However, none of the parameters analyzed in Figure 5 were differentially affected by the individual knockouts. This was established by calculating the differences in parameters' values between each knockout and the corresponding WT control and then conducting independent paired t-tests for these differences at each flash intensity condition (data not shown).

## Discussion

G protein-mediated signaling pathways play a critical role in generating light-responses of photoreceptors and ON-bipolar cells. The rates at which G proteins are activated and inactivated in these cells are major contributing factors in setting the temporal resolution of visual processing. RGS9 and R9AP, along with Gβ5, comprise the GTPase-activating complex for transducin, regulating deactivation of the phototransduction cascade in rods and cones. Recent studies demonstrated that R9AP is also expressed in dendritic tips of ON-bipolar cells [19], [20], suggesting that R9AP, along with its partners RGS11 and Gβ5, might be a component of another GTPase-activating complex that regulates the kinetics of light responses in ON-bipolar cells. One argument supporting this hypothesis is the presence of a distinct, though small, delay in the rising phase of ERG b-waves observed in R9AP^−/−^ and RGS11^−/−^ mice [20], [32], [33], [38].

The first major result obtained in our study is that the b-wave onset delay previously reported in *R9AP* ^−/−^ mice is also present in *RGS9* ^−/−^ mice, despite the lack of documented RGS9 expression in bipolar cells. The phenotypes observed in these animals are very similar in each quantitative characteristic we analyzed, leading us to suggest that the delay may have a common cellular origin in photoreceptors where both proteins are present. Our second finding is that a delayed onset of the b-wave in both mouse types is not followed by slower kinetics of the b-wave rising phase. Instead, this slope in *R9AP* ^−/−^ and *RGS9* ^−/−^ mice is steeper than in WT controls at bright flash intensities. The current theory on the light signal propagation (see Introduction) suggests that a reduction in the GTPase activity of G_o_ due to the lack of RGS11·Gβ5·R9AP expression in ON-bipolar cell dendrites could decrease rather than increase this slope. Thus this finding, though indirectly, also argues against the bipolar cell origin of b-wave delays in both knockouts.

The assumption that the b-wave delay in *RGS9* ^−/−^ and even *R9AP* ^−/−^ mice originates in photoreceptors begs the question of whether it is caused by a slow response recovery in outer segments containing the bulk of both proteins. RGS9 knockout slows the photoresponse recovery phase without changing the rising phase kinetics. Detailed analysis of dim-flash rod photoresponses of *RGS9* ^−*/*−^ mice showed no deviation from the control WT trajectory for at least 100 ms after the flash when the recovery begins [7]. In contrast, both our data (Figures 4 and 5) and the data by Jeffrey et al. [20] clearly indicate that the delay in b-wave onset becomes prominent at the times shorter than 100 ms after the flash. This suggests that, at least in the case of rods responding to dim flashes, the b-wave delay is likely to originate outside outer segments. In fact, small but detectable fractions of both R9AP and RGS9 proteins reside in other photoreceptor compartments [14], [18], [39]. Yet, the mechanism by which R9AP and/or RGS9 may affect photoreceptor synaptic output remains completely unknown. One possibility is that RGS9 and R9AP control the GTPase activity of another G protein regulating synaptic transmission. For instance, the GPCR dopamine D4 receptor was implicated in light- and dark-adaptation of the retina via mechanisms confined to photoreceptors [40], [41]. Currently, no evidence connects D4-mediated G protein signaling with RGS9. However, this may be a productive direction to explore, particularly because in the central nervous system another splice isoform of RGS9 regulates G protein signaling downstream from D2, a dopamine receptor of the same subfamily as D4 [42], [43], [44].

A similar idea that a photoreceptor-specific protein may have different signaling functions in different cellular compartments was previously suggested for recoverin [45]. Recoverin is a Ca^2+^-binding protein [46] thought to regulate rod and cone photoresponse recovery and adaptation by interacting with rhodopsin kinase in the outer segment [47], [48], [49]. However, recoverin is also present in every other compartment of the photoreceptor cell [50]. Parallel recordings from photoreceptors and ON-bipolar cells of recoverin knockout mice revealed that this knockout affected bipolar cell responses significantly earlier than it affected the photoresponse kinetics in outer segments [45]. The authors concluded that recoverin has a second site of action in the photoreceptor synaptic terminals.

There is another piece of puzzle required to be considered in regards to the origin of the b-wave delay: the delay is also observed in the knockout of RGS11, the established R9AP partner in ON-bipolar cells [20], [32], [33], [38], and in the mouse in which RGS7 function was altered by hypomorphic deletion [32], [33]. Though it could not be completely ruled out that small fractions of RGS11 and RGS7 reside in photoreceptors [39], immunolocalization results argue that it is not the case [19], [21], [32], [33], [38], including the study in which the specificity of immunostaining was established with the RGS11 knockout control [19]. Therefore, the b-wave delay phenotype caused by RGS11 knockout and the RGS7 mutant may be potentially caused by different mechanisms than the phenotype observed in *RGS9* ^−/−^ and *R9AP* ^−/−^ mice. To complicate matters even further, single cell recordings from ON-bipolar cells in retina slices of *RGS11* ^−/−^ mice did not reveal any delay in light response kinetics [19]. This suggests that, at least in the case of RGS11 knockout, the manifestation of delayed ON-bipolar cell responses requires the preservation of specific conditions maintained in vivo, which is lost in the retina slice.

Finally, we should stress that the results obtained in this study do not contradict the hypothesis that rapid generation of light responses in ON-bipolar cell dendrites relies on the interchangeable function of RGS7 and RGS11. Rather, we suggest that the specific phenotype of b-wave delay may reflect directly or indirectly modified synaptic output from photoreceptors, or a similar phenotype may originate from not immediately connected presynaptic or postsynaptic mechanisms. Elucidating the function of RGS7 and RGS11 in bipolar cells awaits a more direct analysis of the true RGS7 knockout and the double knockout of both proteins.

## Materials and Methods

### Animals

Mice were handled following the protocol (protocol registry number A041-11-02) approved by the Institutional Animal Care and Use Committees of Duke University and reared under the normal diurnal cycle. C57/Bl6 WT mice were purchased from Charles River. *R9AP* ^−/−^ mice are described in Keresztes et al. [15], and *RGS9* ^−/−^ mice are described in Chen et al. [7]. Wild type (*R9AP* ^+/+^ and *RGS9* ^+/+^, called “WT” throughout the text for simplicity), and knockout mice (*R9AP* ^−/−^ and *RGS9* ^−/−^) were littermates obtained by breeding of *R9AP* ^+/−^ and *RGS9* ^+/−^ mice.

### Immunohistochemistry

Co-immunostaining of R9AP and PKCα in retinal cross sections was performed essentially as described in Herrmann et al. [51] with two modifications: we obtained retinal cross sections from eyecup preparations and used short fixation times of 15 min. For immunostaining of dissociated bipolar cells, we followed a protocol modified from Suzuki et al. [52]: retinas were incubated for 30 minutes at 37°C in standard mammalian saline (135 mM NaCl, 5 mM KCl, 1 mM MgCl_2_, 2 mM CaCl_2_, 10 mM HEPES and 10 mM glucose, pH 7.4) containing 40 U/ml activated papain (Worthington, Freehold, NJ). Digested retinas were rinsed with standard mammalian saline containing 0.1 mg/ml bovine serum albumin (Sigma, St Louis, MO), and the retinal pieces were mechanically triturated with a fire polished glass pipette. Concanavalin-A coated cover glass was prepared by spotting 1 mg/ml concanavalin-A (Sigma, C2010, St Louis, MO) solution in 1 M NaCl on the cover glass for 20 min, followed by rinsing with distilled water and drying via aspiration. Two drops of cell suspension were placed on concanavalin-A coated cover glass preloaded with 100 µl of standard mammalian solution, and the cells were allowed to adhere at 4°C for 1 h. Cells were rinsed with standard mammalian solution to remove non-adhered cells, fixed with 4% PFA in PBS for 15 min, rinsed three times with PBS and further processed following the procedure for immunohistochemistry of retinal cross sections.

For immunostaining of retina flat mounts, retinas were removed from eyecups and fixed for 15 min in 4% formaldehyde. Retinas were rinsed for 15 min in PBS, incubated at 4°C in 15% and 30% sucrose (for 3 h each), blocked for 1 h with goat serum and incubated with a mixture of primary antibodies for 5 days. Retinas were washed 3 times with PBS for 15 min, incubated in secondary antibody solution for 48 h, washed 3 times with PBS for 15 min, and mounted under glass coverslips. Images were acquired by scanning a single optical section through the outer plexiform layer in which R9AP staining was most intense.

Primary antibodies used were rabbit anti-R9AP (1∶200, [14], mouse anti-PKCα (1∶500, Santa Cruz), and anti-peanut agglutinin lectin (PNA) antibody tagged with Alexa-488 fluorophore (1∶250, Molecular Probes); secondary antibodies were goat anti-mouse Alexa Fluor 594 and goat anti-rabbit Alexa Fluor 488 (both 1∶500, Invitrogen).

### Electron microscopy

Transmission electron microscopy in 65 nm-thick retina cross-sections was performed as described in Petters et al. [53].

### Electroretinography

ERGs were recorded using the Espion E^2^ system (Diagnosys LLC, Littleton, MA) as described previously [51]. Mice were dark-adapted overnight, and ERG responses were evoked by a series of 10 flashes ranging from 0.0001 cd·s/m^2^ to 1000 cd·s/m^2^. For flashes up to 0.1 cd·s/m^2^, responses of 10 trials were averaged. For 0.5 and 1 cd·s/m^2^ flash responses, 3 trials were averaged. For brighter stimuli, responses to single flashes were recorded without averaging. Intervals between individual flashes were chosen to ensure that the retinas of *R9AP* ^−/−^ and *RGS9* ^−/−^ mice recovered completely from each flash; specifically, no indications of flash-induced reduction of response amplitudes, enlargement of oscillatory potentials or shortening of implicit times were observed. Based on these criteria, the inter-flash interval times were 10 sec for flashes covering intensities of 0.0001 – 0.005 cd·s/m^2^, 30 sec for flashes covering 0.01 – 0.5 cd·s/m^2^, and 60 sec for the 1 cd·s/m^2^ flash. Recovery times were 120 sec after the 1 cd·s/m^2^ flash, 150 sec after the 10 cd·s/m^2^ flash, and 300 sec after the 100 cd·s/m^2^ and 1000 cd·s/m^2^ flashes, respectively.

### Data analysis

Determination of a-waves and b-wave amplitudes was performed as described [51] using MATLAB software (Version R2007a, Mathworks Inc.). Rod-driven a-waves were measured at 8 ms after the flash stimulus was applied (to exclude post-receptoral contribution from the analysis, e.g. [54]). For b-wave amplitude determination, the oscillatory potentials were removed from the signals by 55 Hz FFT low-pass frequency filtering, and the b-wave amplitude was calculated from the bottom of the a-wave response to the b-wave peak.

Data points from b-wave stimulus-response curves were fitted by Equation 1 using a least-square fitting procedure: 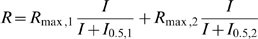
(1)


The first term of Equation 1 is thought to describe rod-mediated responses (index 1), and the second term is thought to describe primarily cone-mediated responses (index 2) observed at the flash intensities ≥1 cd·s/m^2^ for dark-adapted mice [51], [55], [56]. *R_max,1_* and *R_max,2_* are maximal response amplitudes, and *I_0.5,1_* and *I_0.5,2_* are half-saturating flash intensities. Data points from a-wave stimulus-response curves were fitted to a single term of Equation 1.

To examine the b-wave rising phase kinetics, we analyzed three parameters: the time of b-wave onset, the maximal slope of the b-wave rising phase, and the time required to reach the maximal slope of the b-wave rising phase. The b-wave onset was determined from the response traces by visual inspection. The other two parameters for b-waves evoked by flashes up to 0.01 cd·s/m^2^ were determined by obtaining the first derivative of the filtered b-wave response as described before [20]. Responses evoked by flashes ≥0.1 cd·s/m^2^ had considerable a-wave contributions which obscure the filtering procedure; therefore, the a-wave contribution was first subtracted from the ERG trace by fitting the a-wave leading edge to the ‘P3 model’ [57], including the data up to 80% of the a-wave peak [54]. For the purpose of this fitting the maximal a-wave amplitude was set at the actual maximal measured value. Following this procedure of a-wave subtraction, the isolated b-wave response was filtered and the first derivative was calculated as above.

The relation between the time of b-wave onset and flash intensity was fitted to Equation 2:
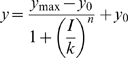
(2)


where *y_max_* is the maximal value, *I* is flash intensity, *n* is the Hill coefficient, and *k* and *y_0_* are fitting parameters. The same equation was used to fit the dependency of the time to the maximal b-wave slope on flash intensity.

The dependency of the maximal b-wave slope value on flash intensity was fitted to Equation 3:



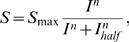
(3)


where *S* is the value of the maximal slope, *S_max_* is the asymptotic value of the maximal slope at saturating flash intensities, *I* is the flash intensity, *I_half_* is the half-saturating flash intensity, and *n* is the Hill coefficient.
